# Development of abnormalities at the neuromuscular junction in the SOD1-G93A mouse model of ALS: dysfunction then disruption of postsynaptic structure precede overt motor symptoms

**DOI:** 10.3389/fnmol.2023.1169075

**Published:** 2023-05-19

**Authors:** Jayne McIntosh, Imane Mekrouda, Maryam Dashti, Claudiu V. Giuraniuc, Robert W. Banks, Gareth B. Miles, Guy S. Bewick

**Affiliations:** ^1^Institute of Medical Sciences, University of Aberdeen, Aberdeen, United Kingdom; ^2^School of Biosciences, Cardiff University, Cardiff, United Kingdom; ^3^Department of Biosciences, Durham University, Durham, United Kingdom; ^4^School of Psychology and Neuroscience, University of St Andrews, St Andrews, United Kingdom

**Keywords:** neuromuscular junction, motor neuron disease, neuromuscular transmission, SOD1-G93A, ALS, acetylcholine receptor, mouse

## Abstract

**Introduction:**

The ultimate deficit in amyotrophic lateral sclerosis (ALS) is neuromuscular junction (NMJ) loss, producing permanent paralysis, ultimately in respiratory muscles. However, understanding the functional and structural deficits at NMJs prior to this loss is crucial for therapeutic strategy design. Should early interventions focus on reversing denervation, or supporting largely intact NMJs that are functionally impaired? We therefore determined when functional and structural deficits appeared in diaphragmatic NMJs relative to the onset of hindlimb tremor (the first overt motor symptoms) *in vivo* in the SOD1-G93A mouse model of ALS.

**Materials and methods:**

We employed electrophysiological recording of NMJ postsynaptic potentials for spontaneous and nerve stimulation-evoked responses. This was correlated with fluorescent imaging microscopy of the postsynaptic acetylcholine receptor (AChR) distribution throughout the postnatal developmental timecourse from 2 weeks to early symptomatic ages.

**Results:**

Significant reduction in the amplitudes of spontaneous miniature endplate potentials (mEPPs) and evoked EPPs emerged only at early symptomatic ages (in our colony, 18-22 weeks). Reductions in mEPP frequency, number of vesicles per EPP, and EPP rise time were seen earlier, at 16weeks, but this reversed by early symptomatic ages. However, the earliest and most striking impairment was an inability to maintain EPP amplitude during a 20 Hz stimulus train, which appeared 6 weeks before overt *in vivo* motor symptoms. Despite this, fluorescent *α*-bungarotoxin labelling revealed no systematic, progressive changes in 11 comprehensive NMJ morphological parameters (area, shape, compactness, number of acetylcholine receptor, AChR, regions, etc.) with disease progression. Rather, while NMJs were largely normally-shaped, from 16 weeks there was a progressive and substantial disruption in AChR concentration and distribution within the NMJ footprint.

**Discussion:**

Thus, NMJ functional deficits appear at least 6 weeks before motor symptoms *in vivo*, while structural deficits occur 4 weeks later, and predominantly within NMJs. These data suggest initial therapies focused on rectifying suboptimal NMJ function could produce effective relief of symptoms of weakness.

## 1. Introduction

The fundamental deficit of amyotrophic lateral sclerosis (ALS) is the loss of functional neuromuscular junctions (NMJs), producing the weakness and paralysis, and ultimate respiratory arrest, characteristic of the disease. Since 95% of all ALS occurs spontaneously, prophylactic treatments are not likely to be applicable to most patients. Thus, treatment will likely start when symptoms first appear, at least for the foreseeable future and for most sufferers. To design optimal treatments after onset of disease symptoms, it is important to know the condition of a patient’s NMJs at that stage, i.e., to understand what precise issues need to be addressed by the therapy/therapies. For example, whether the earliest symptoms are due largely to the complete removal of NMJs, or more the result of generally impaired function in sick but still viable NMJs. Further, if the latter, it is important to understand how much structural disruption drives the loss of function, or whether the functional loss occurs prior to large-scale morphological disruption. These issues are important for informing the design of treatment therapies and evaluating their likelihood of success. For example, whether the treatment should focus on re-innervation of NMJs that have been withdrawn or enhancing function in NMJs that are still present but functionally impaired. It would seem intrinsically easier and potentially offer hope of more effective symptomatic relief to enhance function in NMJs that are still partly intact, rather than a need to entirely re-establish NMJs that have been completely withdrawn. Finally, if the NMJs are disrupted, it is important to understand if this is secondary to impaired pre-synaptic function, or if postsynaptic structural disruption prevents effective transmission. Again, this will inform the search for the location of the most suitable therapeutic target. While there are many previous studies of NMJs, in both structure and function, in ALS disease models (Alhindi et al., 2022), most look at either structure, or function but not both. Importantly, none have correlated functional and structural changes of the NMJ in the same muscle. Most look at 2–4 timepoints in disease progression. None have looked at function throughout postnatal development. Finally, none have examined the appearance of functional impairment under *in vivo*-like patterns of use, i.e., during the trains of stimuli that most often activate NMJs in life (Prakash and Sieck 1998; Prakash et al., 1999). Therefore, to address these questions we have mapped the postnatal development of functional and structural deficits at NMJs from 2 weeks of age to the onset of the first overt motor symptoms (hindlimb tremor) *in vivo* in the SOD1-G93A mouse model of ALS, which over-expresses the defective human SOD1 gene (hSOD1-G93A). We suggest the appearance of motor symptoms in the mouse model is likely to be equivalent to the point at which most patients would present for diagnosis and treatment. Because the major cause of death is respiratory failure (Kiernan et al., 2011), the main respiratory muscle, the diaphragm, was studied.

Information on the detailed time course of denervation and associated functional changes specifically in the diaphragm in hSOD1-G93A models of ALS is lacking as most studies examine only at particular, strategic timepoints (Cappello et al., 2012). Moreover, there are no previous studies with high temporal precision regarding the postnatal time course of correlated changes in NMJ structure and function up to the appearance of the first motor symptoms. However, in their detailed structural study of 435 NMJs at early symptomatic stages (again defined by appearance of hindlimb tremor), Schaefer et al. (2005) report in another thoracic muscle (sternomastoid) that, 92.6% of the NMJs are normally innervated structurally, pre-and postsynaptically; 2.5% are denervated (1.6% with some disrupted AChR labeling intensity), 0% had fragmented axons, 4.4% have thin axons (0.5% with faint AChR labeling in places). Thus, 2.1% had disrupted AChR distributions. However, neither group performed functional studies of transmission even at this specific timepoint. Thus, there has been no previous study correlating the emergence of NMJ structure/function perturbations in this muscle, to investigate the importance of each and their relative contributions to functional deficits.

In the present study, function was determined by sharp-electrode postsynaptic recording of spontaneous and evoked release of neurotransmitter, quantal analysis of release and ability to maintain release during functionally relevant trains of stimuli (2 min at 20 Hz) (Hennig and Lomo, 1985; Prakash et al., 1999). In the same muscles, after recording, postsynaptic acetylcholine receptors (AChRs) were labeled with fluorescent α-bungarotoxin, and 11 postsynaptic morphological parameters analyzed, including shape, dimensions, complexity, and fraction of the NMJ occupied by AChRs. In addition, the intensity and fine pattern of AChR distribution within individual NMJs were quantified. The data suggest the first neurotransmission deficits are in the ability to maintain release, and they appear 6 weeks before overt motor issues are present. These are followed by a reduction in expression and redistribution of AChRs within the NMJ, rather than large-scale shape change. These data suggest presynaptic impairments precede, and therefore drive, the structural disruption. Therefore, early therapies might best be aimed at enhancing functional transmission of impaired but intact NMJs, and that prompt treatment might be able to prevent progression toward, or even reverse existing, structural deficits.

## 2. Materials and methods

### 2.1. Scoring system for disease progression

The scoring system used to assess the severity of ALS in the hSOD1-G93A mouse was that designed by Weydt et al. (2003) as follows.

4 = normal, no sign of motor dysfunction

3 = hind limb tremors evident when raised by the tail

2 = gait abnormalities

1 = dragging of at least one hind limb

0 = inability to right itself within 30 s after being placed on its back.

The oldest animals used in the present study were stage 3, which we termed ‘early symptomatic’, and in our colony were aged 18–22 weeks of age (126–154 days). As this is the earliest detected sign of motor abnormality, we suggest this probably corresponds to the earliest stage at which patients present for treatment.

### 2.2. Sharp electrode recording of neuromuscular transmission

#### 2.2.1. Animals and dissection

All experiments were carried out at the University of Aberdeen and approved by the Animal Welfare and Ethical Review Board, and Home Office (HO) license to GSB. Animals were kept in standard husbandry conditions within HO guidelines (group housed, cardboard tube and climbing rod environmental enrichment, bedding of corncob, wood shavings and paperwool, changed weekly, at 19–21°C, 45–65% relative humidity, 12 h dark:12 h light cycle, *ad libitum* access to standard chow and drinking water). All mice were checked daily. ARRIVE guidelines were followed except that blinding and full randomization were not possible. The HO license required all early symptomatic animals to be used within 24 h of hindleg tremor being detected. Thus, experiments on symptomatic hSOD1-G93A mice occurred within 24 h of animal facility notification after their daily checks. Pseudorandomization of age and genotype occurred by default, as animal usage was determined by small litter size (usually <6), date of birth/usage, only 1 animal (2 hemidiaphragms) maximum per day, experimentation only on weekdays, age-and littermate matching, and within the ±2 days of age required, and equalizing age group sizes. hSOD1-G93A mice founders were male B6SJL-TgN (SOD1-G93A)1Gur/J (SOD1G93a) from the University of St Andrews colony, that were established and maintained on a congenic C57bl/6 background strain from the University of Aberdeen colony. hSOD1-G93A and wildtype mice from the same litter (see Table 1; Age range, weight range and number of animals used for electrophysiology) were killed by cervical dislocation [Schedule 1 approved method, Animals (Scientific Procedures) Act 1986 incorporating European Directive 2010/63/EU]. Mice were killed at 2, 4, 8, 12, 16, or 20 weeks (wild-type mice) (±2 days) or when motor symptoms first appeared (hSOD1-G93A mice), which was 18–22 weeks of age. All mice came from the same colony, and in the great majority of cases the wild-type mice were littermates of the hSOD1-G93A mice of the corresponding age. The genotype of mice was determined at 2 weeks, when each mouse was given an unique identifying ear clip.

**Table 1:** Age range, weight range, and number of animals used for electrophysiology.

Wildtype	Age range	Weight range (*g*)	No. Animals
2 weeks	±2 days	6–9	6
4 weeks	±2 days	7–17	6
8 weeks	±2 days	19–28	5
12 weeks	±2 days	20–29	7
16 weeks	±2 days	29–35	5
20 weeks	Symptomatic age-match	20–32	8
**hSOD1-G93A**			
2 weeks	±2 days	6–9	6
4 weeks	±2 days	12–16	7
8 weeks	±2 days	18–28	9
12 weeks	±2 days	19–28	13
16 weeks	±2 days	18–30	8
18–22 weeks	Early symptomatic	17–27^***^	16

Animal weight generally increased with age, except early symptomatic hSOD1-G93A mice, which were lighter than WT littermates (***p < 0.001, Student’s *t*-test).

The phrenic nerve on each side was separated from the mediastinum, transected as high as possible in the thoracic cavity and removed in continuity with the diaphragm. This gave a total length of 1–2 cm (increasing with age, but constant at any age). The diaphragm was split into two hemi-diaphragm/nerve preparations down the midline, and pinned out on PDMS (Sylgard 184, Dow Corning, Stade, Germany) lined 35 mm diameter tissue culture dish at room temperature under gassed (95% oxygen, 5% carbon dioxide) physiological saline solution containing (mM): 138.8 NaCl, 4 KCl, 12 NaHCO_3_, 1 KH_2_PO_3_, 1 MgCl_2_, 2 CaCl_2_, 11 glucose (Liley, 1956). Dissections and recordings were performed at room temperature (18–22°C) and the time from initiation of dissection to initiation of recording was 2–3 h, and at any particular age the nerve stump length was constant, although absolute length increased with age. Under these conditions, no discernible deterioration of NMJ structure or function is observed in healthy, WT mice (Brown et al., 2015).

#### 2.2.2. Electrophysiological recording

The nerve was taken into the suction electrode and stimulated with a gradually increasing voltage (0–10 V, 0.2 ms duration) using a Master 8 pulse pattern generator delivered through an Isoflex stimulus isolation unit (Intracel, Royston, United Kingdom). The health of the preparation was determined by the minimum stimulus to evoke a twitch (<1 V) and to produce the maximum visually assessed twitch (2.4–5 V). For stable electrophysiological recordings from NMJs, nerve-evoked muscle contraction was then blocked by bath application with at least 2 μM μ-conotoxin GIIIB (Alomone Labs, Jerusalem, Israel) for 55 min, which was the minimum concentration and duration to achieve total paralysis in healthy adult preparations in response to a nerve stimulation intensity of double the previously determined maximum. The toxin exposure was extended, and then if necessary applied at a higher concentration, for muscles from mice of both genotypes at 2–4 weeks and also at early symptomatic stages in hSOD1-G93A, up to a maximum of 8 μM and 120 min. All muscles were tested at the shortest time and lowest concentration (2 μM for 55 min) before toxin exposure was increased if necessary. Once paralysis was established, toxin was removed to avoid nerve block with excessive incubation.

μ-Conotoxin GIIIB is a highly selective and high affinity antagonist for rodent mammalian skeletal muscle voltage-gated sodium channels (Hill et al., 1996) with no effect on the nerve voltage-gated sodium channels at the concentrations used here (Cruz and Olivera, 1986). This allowed intracellular recordings of normal neurotransmission from NMJs to be made without triggering postsynaptic action potentials and subsequent contraction.

#### 2.2.3. mEPP, EPP, and RMP recording

A sharp glass intracellular microelectrode filled with 3 M KCl was used to record spontaneous miniature end plate potentials (mEPPs), evoked end plate potentials (EPPs) and resting membrane potential (RMP). Electrophysiological recordings used an Axoclamp 2B amplifier (Axon Instrument, Foster City, CA, United States) and were stored onto a computer hard drive using Strathclyde University Electrophysiology Software Whole Cell Program (WCP version 3.5, University of Strathclyde, Glasgow, United Kingdom). Electrode placement was controlled visually under a modified Nikon Optiphot compound microscope (Micro Instruments, Oxford, United Kingdom) using a 3.5x Zeiss 3.2/0.07 (Carl Zeiss Microscopy GmbH, Germany) objective and a free-standing stage. NMJs were located by impaling near one of the first small branches leaving the main nerve trunk as it enters the muscle, with a glass microelectrode. NMJs were identified by the presence of mEPPs, plus an EPP rise time of ≤1.5 ms, indicating the recording electrode is within 200 μm of the NMJ (Auerbach and Betz, 1971). mEPPs were recorded for 30 s. Then 3x EPPs evoked by nerve stimulation at 0.1 Hz stimulation were recorded, to obtain a mean EPP amplitude. This frequency was chosen to minimize EPP rundown with repeated stimulation. Impalements progressively moved along the muscle end-plate band systematically away from the muscle nerve entry point to avoid multiple recordings from the same fiber. Recordings were made until 10 NMJs were recorded or for an hour, whichever was the shorter. The latter was not common but most often affected recordings from early symptomatic hSOD1-G93A mice.

#### 2.2.4. 20  Hz trains

In some muscles. After recording from 10 NMJs, responses to 20 Hz were recorded. A further NMJ impalement was made and the first 30 s of mEPP and 3x EPPs at 0.1 Hz were again recorded. Then, to examine the ability of the terminals to maintain transmitter release, EPP trains were recorded in response to a continuous stimulus train of 20 Hz for 2 min.

### 2.3. Neuromuscular junction imaging

After electrophysiological recording some hemi-diaphragm/phrenic-nerve preparations (See Table 2 Number of animals and muscles used to determine incidence of granularity and extrajunctional AChRs.) were incubated in gassed saline containing 2 μg/ml TRITC α-bungarotoxin for 1 h to label the AChRs, rinsed twice in toxin-free saline before a wash in gassed saline (1 h) to remove unbound toxin.

NMJs were viewed with a Nikon Optiphot compound microscope and a free-standing stage (Micro Instruments, Oxford, United Kingdom) initially with a 3.5x (Zeiss 3.2x, 0.07NA; Carl Zeiss Microscopy GmbH, Germany) objective to locate the end plate band. High magnification images were acquired through a 40x water emersion objective (Zeiss 40x, 0.75NA; Carl Zeiss Microscopy GmbH, Germany), with a Prior HBO 100 Hg lamp illuminator with an excitation/emission filter of 510–560/590 nm (Carl Zeiss MicroImaging GmbH), Shutterhub and Acquisitionhub (Improvision Ltd., United Kingdom), using a Retiga ER (Q-imaging, Canada) digital camera and captured onto a PC hard drive, using Volocity 5.3.1 (Improvision Ltd., United Kingdom). Twenty *en face* NMJs were captured per preparation near the nerve entry point, the region in which electrophysiological recordings were performed. The nerve enters in the center of the muscle, so the first 10 *en face* NMJs were captured moving along the endplate band away from the nerve entry point in one direction, then a further 10 from the nerve entry point in the other direction. This minimized time of data capture and bias to any particular preparation/age/genotype.

### 2.4. Data analysis

#### 2.4.1. Electrophysiology

mEPP and EPP amplitude was measured, and the RMP at which each of these was recorded was noted. This was used in calculations later, as RMP often changed over the duration of the recording at each NMJ. Recordings in which RMP became more positive than −50 mV at any time were rejected. The quantal content of the EPP (number of vesicles released per stimulus, QC) was calculated after correcting EPPs for non-linear summation, with the following formula (McLachlan and Martin, 1981).

QC = E / [M x (1 – f (E/Vm))].

E = amplitude of EPP.

M = mean amplitude of mEPP.

Vm = resting membrane potential.

f = correction factor for non-linear summation (0.8 was used).

To compare mEPP and EPP amplitude between muscle fibers and muscles, they were corrected to -70 mV, using the equation:

M corrected = mEPP (70/V_mEPP_) or EPP (70/V_EPP_), as appropriate.

V_mEPP_/V_EPP_ is the resting membrane potential (RMP) at which the responses were recorded.

##### 2.4.1.1. Statistical analysis

These data are presented as mean ± standard error of the mean (SEM). Analysis of differences between experimental and control groups was carried out using a two-way analysis of variance (ANOVA), followed by individual mean post-tests at specific ages with Bonferroni correction to test for differences between experimental and control groups. A *p-*value of <0.05 was taken as significant.

#### 2.4.2. Neuromuscular junction morphology

For this analysis, as many NMJs as reached criterion of ‘en face’ (<10% obscured/curved around muscle fiber) were analyzed. The Neuro-morph semi-automated workflow developed by (Jones et al., 2016) was used for quantitative analysis of AChR distributions at the NMJ. The 6 core postsynaptic variables delimited were (see also Supplementary Figure S1):

a. Endplate area
b. Endplate diameter
c. Endplate perimeter
d. Acetylcholine receptor (AChR) area
e. AChR perimeter length
f. Number of AChR clusters

The further 4 derived variables from these parameters were:

g. Average AChR cluster area (d/f)
h. Endplate area unoccupied by AChRs (a-d)
i. Compactness ((a/d)*100)
j. Fragmentation (1-(1/f))

The person performing the image analysis was blinded to the genotype of the mice. The accuracy and reproducibility of the semi-automated NMJ-morph algorithm was first verified manually, where the NMJ parameters were compared when determined manually and by different automatic threshold criteria in a sub-set of images from 3 mice at different ages and genotypes. The automatic selection criteria were refined iteratively until manually tracing the NMJ using the drawing tool on ImageJ, produced values <10% different from the semi-automated ‘Huang’ threshold approach. At this point, in fact, the majority of errors were < 5%. Once such minimal discrepancy was routinely achieved between the methods, the NMJ-morph semi-automated algorithm was used to derive the variables of all NMJs (see Supplementary Figure S1 legend for details).

**Figure 1:**
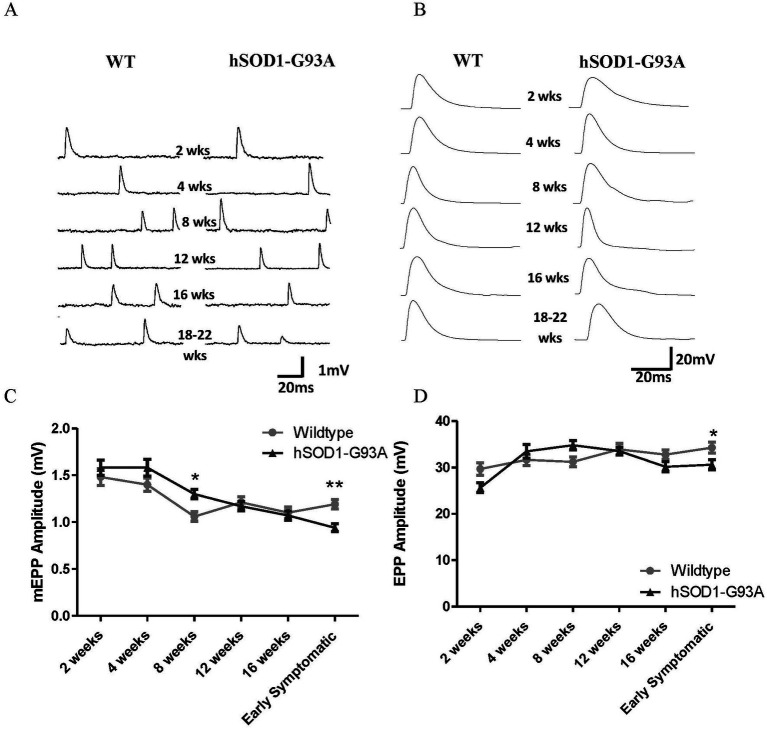
Neuromuscular transmission in hSOD1-G93A mice begins to decline at 8 weeks, and becomes significantly impaired at onset of overt motor symptoms. Representative mEPPs **(A)** and EPPs **(B)** in the muscles at each age. **(C)** mEPP amplitudes in both genotypes decrease with age (*p < 0.0001, Two-way ANOVA). Initially, the mean mEPP amplitude tended to be higher in hSOD1-G93A diaphragms, becoming significantly so at 8  weeks (*p* < 0.05, Two-way ANOVA – Bonferroni post-test). However, the age-dependent decrement was then faster at hSOD1-G93A NMJs, and mEPPs became significantly smaller at early symptomatic stages (**p < 0.01, Two-way ANOVA – Bonferroni post-test). **(D)** In wildtype mice, EPP amplitudes increase throughout postnatal development (*p* < 0.0001, Two-way ANOVA – Bonferroni post-test). In hSOD1-G93A mice, EPP amplitudes begin to decrease at 8  weeks, becoming significantly smaller by early symptomatic stages (*p < 0.05, Two-way ANOVA – Bonferroni post-test). Thus, neurotransmission in disease declines from 8 to 12 weeks and becomes significantly impaired by the early symptomatic stages of disease onset. 30–77 NMJs per time point, from 4 to 10 muscles.

##### 2.4.2.1. Statistical analysis

Many of these data sets were not normally distributed, so analysis was by the non-parametric Ansari-Bradley test, followed by a Mann–Whitney U comparison for differences between medians at particular ages. A *p* < 0.05 was taken as significant.

#### 2.4.3. Fractal analysis of endplate perimeter

The perimeter of the endplate was further analyzed for change of complexity during aging and disease progression, by calculation of the fractal dimension (Mandelbrot, 1977). This quantifies the complexity of a shape into a numerical value, allowing direct quantitative comparisons. It has been applied widely in biomedical sciences, ranging from brain images (Sandu et al., 2014) to single neurons in culture (Smith et al., 2021). We adopted the most widely used, box-counting, method. This covers the AChR perimeter with boxes of increasing size. A “hit” was scored if at least one pixel in the perimeter line contacts the box. The fractal dimension is given by the slope, in a double logarithmic plot, of the number of ‘hits” versus box size. The box sizes were set as 1, 2, 4, 8, 16, 32, 64 pixels per side. Analysis was performed using open-source software (ImageJ, Rasband, W.S., U. S. National Institutes of Health, Bethesda, MD, United States,^1^ 1997–2018). The Fractal Box Count function calculated the perimeter shape complexity, giving values between 1 and 2. The minimum output, 1, is a totally smooth perimeter, whereas values closer to 2 have a more complex perimeter shape.

##### 2.4.3.1. Statistical analysis

These data were not normally distributed, so are presented as median and interquartile range. Statistical comparison was by the non-parametric Ansari-Bradley test, followed by a Mann–Whitney comparison for differences between medians at particular ages. A *p* < 0.05 was taken as significant.

#### 2.4.4. Variability of AChR labeling intensity across the NMJ

A second semi-automated tool was used to analyze variability of labeling intensity within an NMJ. A minimal rectangular selection was made that enclosed the entire NMJ. Again, obvious regions not relevant to the NMJ of interest were excluded manually. Images were converted to 8-bit (0–255 gray levels) and smoothed to remove sporadic pixel intensity maxima or minima. An intensity frequency histogram was then generated for the entire image, and values plotted in Excel. This generated a curve with a bimodal distribution (Supplementary Figure S2A, for wildtype NMJ in Supplementary Figure S1). The large peak of intensity values <40 represents background/non-AChR labeling, and the lower peak (right, higher labeling intensity), represents the AChR labeling. Values <40 were excluded, leaving a frequency curve from 40 to 255. The shallow minimum represents low intensity AChR labeling (Supplementary Figure S2B), and the peak represents higher AChR labeling intensity. The ratio of the minimum to the peak maximum indicated the proportion of the AChR pixels (i.e., area) with reduced labeling intensity. The greater the ratio value, the larger proportion of pixels of the NMJ had reduced labeling. This was repeated for each NMJ.

##### 2.4.4.1. Statistical analysis

Data are presented as mean ± SEM. Differences in mean ratios were assessed by 2-Way ANOVA comparing age and genotype, with a Bonferroni-corrected post-test between genotypes at specific ages. A *p* < 0.05 was taken as significant.

#### 2.4.5. Disruption of AChR labeling pattern

For some muscles at each age, NMJs were analyzed for details of AChR disruption (see Table 2 for number of preparations used for each age group and genotype).

**Table 2:** Number of animals and muscles used to determine NMJ morphology.

	No. Wildtype	No. muscles	No. SOD1-G93A	No. muscles
4 weeks	2	4	5	9
8 weeks	2	4	4	7
12 weeks	3	6	4	8
16 weeks	2	4	3	6
≥18 weeks	2	4	5	10

Two to five mice were used per genotype in each age group. Each animal had 2x hemi-diaphragm muscles and a maximum of 20 NMJs were analysed from each muscle.

All NMJs for which a substantial area of the AChR was observed were categorized by two qualitative parameters. That is, they were not necessarily entirely en face, as this was not an essential requirement for this semi-qualitative analysis. First, whether the AChR distribution displayed an obvious area of granular (floccular) appearance (Supplementary Figure S3A), sub-divided into whether this was <50% or >50% of the total AChR area. The second aspect was the presence of small extra-junctional AChR clusters, indicative of nerve sprouting/AChR removal (Supplementary Figure S3B). The latter were defined as ‘atypical’ small areas of labeling visible beyond the main AChR clusters of the NMJ, i.e., much smaller, and usually obviously dimmer than the bulk of the NMJ.

##### 2.4.5.1. Statistical analysis

Many of these data were not normally distributed, so histogram data for incidence of atypical AChR distributions were analyzed by non-parameteric statistical methods, and data are presented as median ± interquartile range. A *p* < 0.05 was taken as significant.

## 3. Results

### 3.1. hSOD1-G93A mouse colony characteristics

The onset of symptoms in our colony of hSOD1-G93A was delayed relative to the source founders (Broadhead et al., 2022) However, all mice allowed to survive developed symptoms, and consistent with disease onset, body weight (Table 1) was reduced at this early symptomatic stage of ALS progression (*p* < 0.001, Student’s *t*-test). Thus, they developed the usual early symptoms, but at a later date. A lower-than-average body mass has been related with ALS in humans, which may be due to hyper-metabolism that develops in ALS (Turner et al., 2013).

### 3.2. Neurotransmission deficits develop with age in hSOD1-G9A mice

To investigate when transmission deficits appeared in hSOD1-G93A NMJs, various parameters were compared in wildtype and hSOD1-G93A mice at 2, 4, 8, 12, 16 weeks, plus early symptomatic stages (hindlimb tremor first evident) of 18–22 weeks old. Each group was age-matched with wildtype littermates. The parameters, and their potential pathological indication, are given each section.

#### 3.2.1. Miniature endplate potential amplitude is smaller in early symptomatic hSOD1-G9A mice

mEPPs are postsynaptic potentials recorded due to random release of a single vesicle of neurotransmitter from the presynaptic terminal. The amplitude gives a measure of efficacy of ACh release in activating the muscle. To investigate whether spontaneous mEPP transmission deficits appeared in hSOD1-G93A NMJs, mEPP amplitudes were compared across the full age-range in wildtype and hSOD1-G93A mice, i.e., at 2, 4, 8, 12, 16  weeks, plus early symptomatic stages (hindlimb tremor first evident) of 18–22 weeks old, which were age-matched with wildtype littermates. Typical mEPPs at each age are shown in Figure 1A. In wild-type, consistent with the previous studies in the rat for soleus and extensor digitorum longus (EDL) muscles (Bewick et al., 2004) and diaphragm muscles (Kelly, 1978) mEPP amplitudes decreased with age (*p* < 0.0001, Two-way ANOVA), beginning at 1.48 ± 0.09 mV (37 NMJs, 4 muscles) at 2 weeks and reducing to 1.19 ± 0.05 mV (63 NMJs, 7 muscles) at 18–22 weeks. This reflects the growth in diameter, and hence decrease in input resistance, of the postsynaptic muscle fiber (Bewick et al., 2004).

In hSOD1-G93A mice, initially there is a trend for mEPP amplitudes to be larger than in wildtype mice of the same age (Figure 1C), becoming significant at 8 weeks old (1.30 ± 0.5 mV, *n* = 72, eight muscles) compared to WT (1.06 ± 0.1 mV, *n* = 47, 5 muscles; *p* < 0.05, Bonferroni post-test). At 12 and 16 weeks, mEPP amplitudes are the same as in wildtype mice. However, in early symptomatic hSOD1-G93A mice, mEPP amplitudes had become significantly smaller (0.94 ± 0.04 mV, *n* = 77, 8 muscles) than in age-matched wildtype controls (1.19 ± 0.1 mV, *n* = 63, 7 muscles; *p* < 0.01, Bonferroni post-test), a reduction of 21.0%.

#### 3.2.2. Endplate potential amplitude is smaller in early symptomatic hSOD1-G9A mice

Endplate potentials result from the simultaneous release of many vesicles, which in healthy NMJs is enough to depolarize the muscle fiber to threshold for muscle fiber contraction. It seems likely that an early indicator of disease in motor terminals would be impaired neurotransmitter release, and so decreased EPP amplitude. This would reduce the likelihood of reaching threshold for triggering contraction, which might be a contributory factor to the symptoms of muscle weakness in ALS. Typical EPPs for wildtype and hSOD1-G93A are shown in Figure 1B. In wildtype, EPP amplitudes actually increased throughout postnatal development (Figure 1D; *p* < 0.0001, Two-way ANOVA), again consistent with studies in rat muscles (Kelly, 1978; Bewick et al., 2004), and reflecting an increase in transmitter release per stimulus. In the hSOD1-G93A mice, initially EPP amplitudes also increased with age. However, beyond 8 weeks old, EPP amplitudes started to decline, becoming significantly smaller than at wildtype NMJs at early symptomatic ages (34.3 ± 1.2 mV, *n* = 63, 7 muscles vs. 30.6 ± 1.1 mV, *n* = 77, 8 muscles, *p* < 0.05 Bonferroni post-test).

#### 3.2.3. Number of vesicles per EPP is reduced at 16 weeks but recovers in symptomatic hSOD1-G93A mice

**Figure 2:**
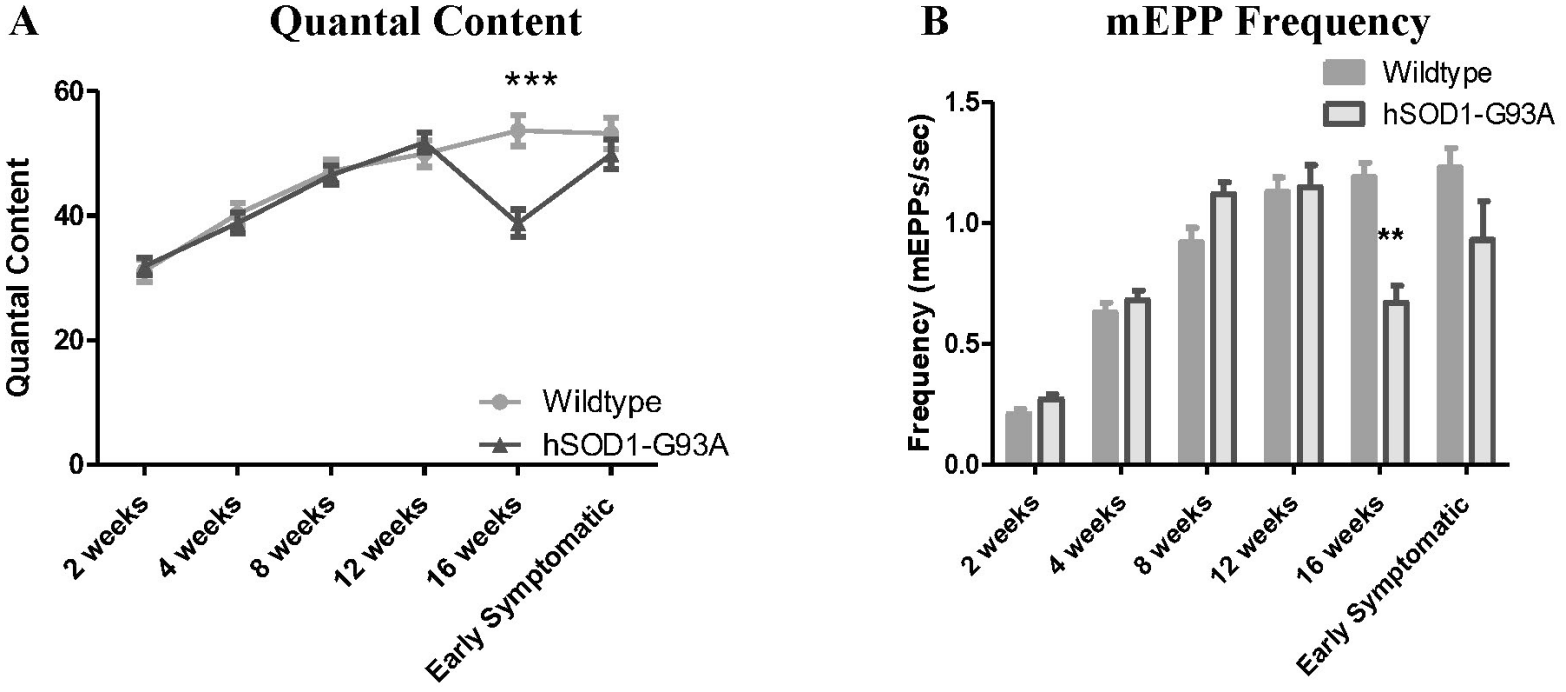
hSOD1-G93A mice have reduced quantal content (QC) and mEPP frequency at 16 weeks old, and increased EPP rise time at 12 weeks. **(A)** QC increased with age in hSOD1-G93A mice and their wildtype counterparts over most of the developmental time course. It briefly reduced at 16 weeks old (***p < 0.001, Two-way ANOVA – Bonferroni post-test), but recovered again by early symptomatic stages. **(B)** mEPP frequency increased with age until 8 weeks, then stabilized in WT NMJs. At hSOD1-G93A NMJs, mEPP frequency was normal until 16 weeks, when it was transiently reduced (**p < 0.01, Two-way ANOVA – Bonferroni post-test).

EPP amplitude is a function of the number of vesicles (mEPPs) being released per nerve stimulus. This is another important measure of the health and efficacy of a motor nerve terminal. The actual number of vesicles released per action potential (quantal content, QC) was calculated to better understand the timing and effects of disease progression. In wildtype mice, as in previous rat studies, QC increased with age. In hSOD1-G93A mice, QC was not significantly different from wildtype at most ages (Figure 2) but there was a marked reduction at 16 weeks (by 27.7%, *p* < 0.001, Two-way ANOVA – Bonferroni post-test). However, despite the decrease in mEPP and EPP amplitudes (Figure 1), the QC then recovered to wild-type levels by early symptomatic stages. Thus, the EPP amplitude seems to fall in diseased motor terminals because they fail to increase the number of vesicles released to compensate adequately for the large decrease in mEPP size.

#### 3.2.4. Reductions in mEPP frequency at 16  weeks and EPP rise time at 12  weeks recover in symptomatic hSOD1-G93A mice

We next examined other electrical parameters, both pre-and post-synaptically: mEPP frequency, EPP rise time, and NMJ resting membrane potential. mEPP frequency is a presynaptic property that often correlates directly with probability of release. EPP rise time (rate of depolarization) can indicate disrupted synchronicity of release, alterations in activity of the AChesterase breaking down ACh in the NMJ synaptic cleft. Reduced (less negative) resting membrane potential can indicate the disease directly affecting the muscle, or inadequate innervation. In wild-type NMJs, mEPP frequency increased up to 12 weeks old when it plateaued. In hSOD1-G93A mice, as with QC, mEPP frequency was normal until 16 weeks, when it was significantly reduced by 43.7%; *p* < 0.01, Two-way ANOVA – Bonferroni post-test (Figure 2) but again recovered at early symptomatic stages. All of these changes in mEPP frequency correlate well with the changes in QC, and the relationship with probability of release stated above.

**Figure 3:**
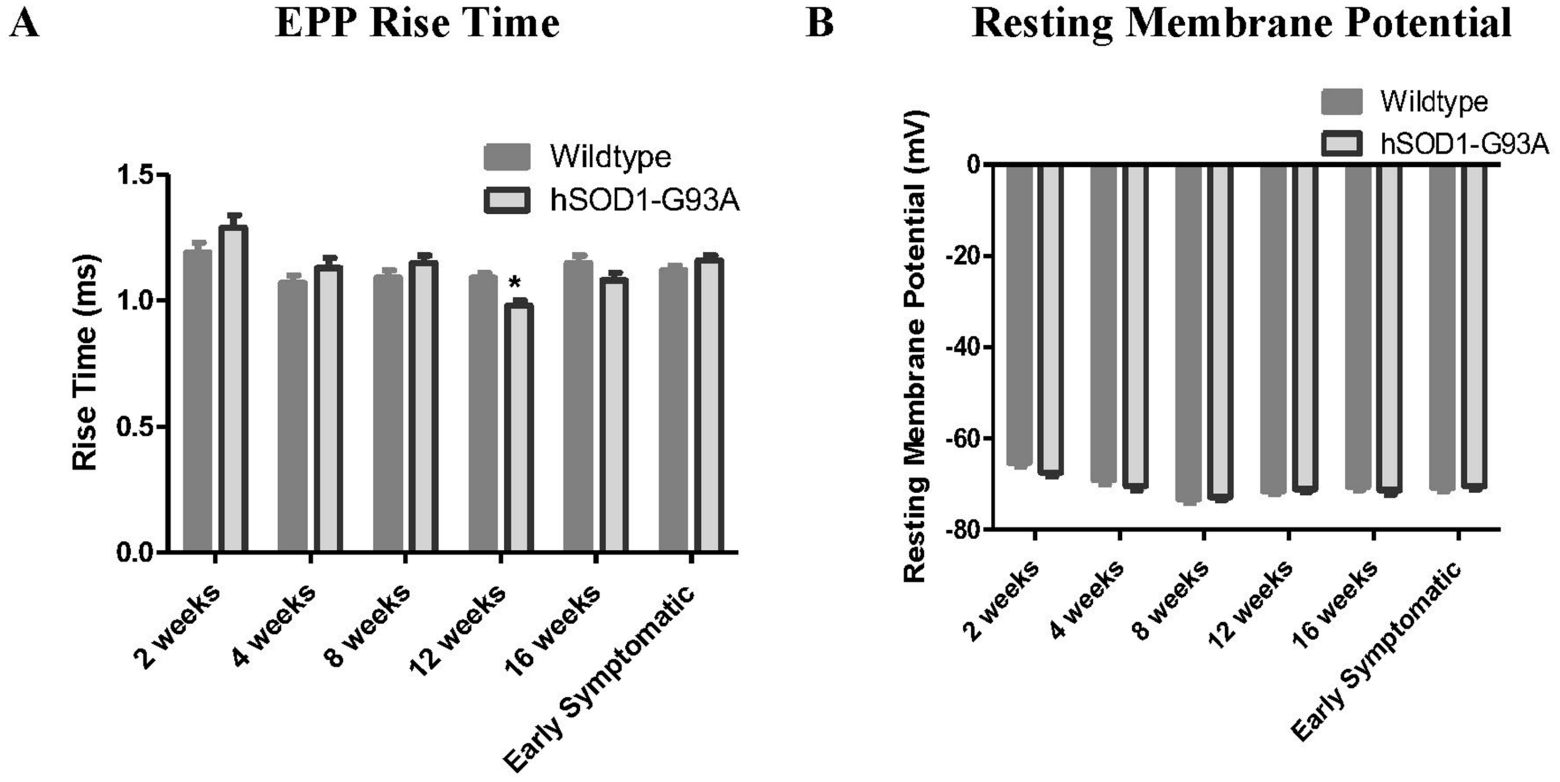
hSOD1-G93A mice transiently exhibit reduced EPP rise time. **(A)** EPP rise time was constant with age in WT, but transiently became faster at 12 weeks old (*p < 0.05, Two-way ANOVA – Bonferroni post-test) in hSOD1-G93A. **(B)** Mean RMP was constant throughout postnatal development, and showed no significant differences in hSOD1-G93A.

EPP rise time remained effectively constant throughout development in wild-type muscles. Interestingly, in hSOD1-G93A, the EPP rise time was significantly faster at 12 weeks old (*p* < 0.05, Two-way ANOVA – Bonferroni post-test; Figure 3A). Although rise times returned to normal thereafter, it seemed that at early symptomatic stages there was more difficulty in finding EPPs within our selection criteria of focal recordings (i.e., <1.5 ms for 0–100% rise time). Slower EPPs are excluded from the analysis, therefore. Regardless, the significantly fast rise-times at 12 weeks indicates transmission is undergoing re-organization at this early time.

RMP in wildtype mice did not change significantly during postnatal development and, at least for muscle fibers with mEPPs and EPPs, RMP in hSOD1-G93A was not significantly different (Figure 3B; *p* > 0.05, Two-way ANOVA – Bonferroni post-test).

#### 3.2.5. hSOD1-G93A NMJs have impaired ability to maintain EPP amplitude during stimulation trains from 12  weeks onwards

Responses to single stimuli are indicative of fundamental neurotransmitter release characteristics experimentally. However, NMJs are normally active *in vivo* with trains of stimuli. Indeed, it seems likely that the ability to maintain effective neuromuscular transmission during trains of stimuli is the most important functional requirement. Thus, we next investigated how the ability to maintain release during repetitive activity might be affected by disease, and if so, when. Twenty Hz stimulation was chosen as this is the *in vivo* activation rate of slow motor units in rats (Hennig and Lomo, 1985; Prakash et al., 1999).

**Figure 4:**
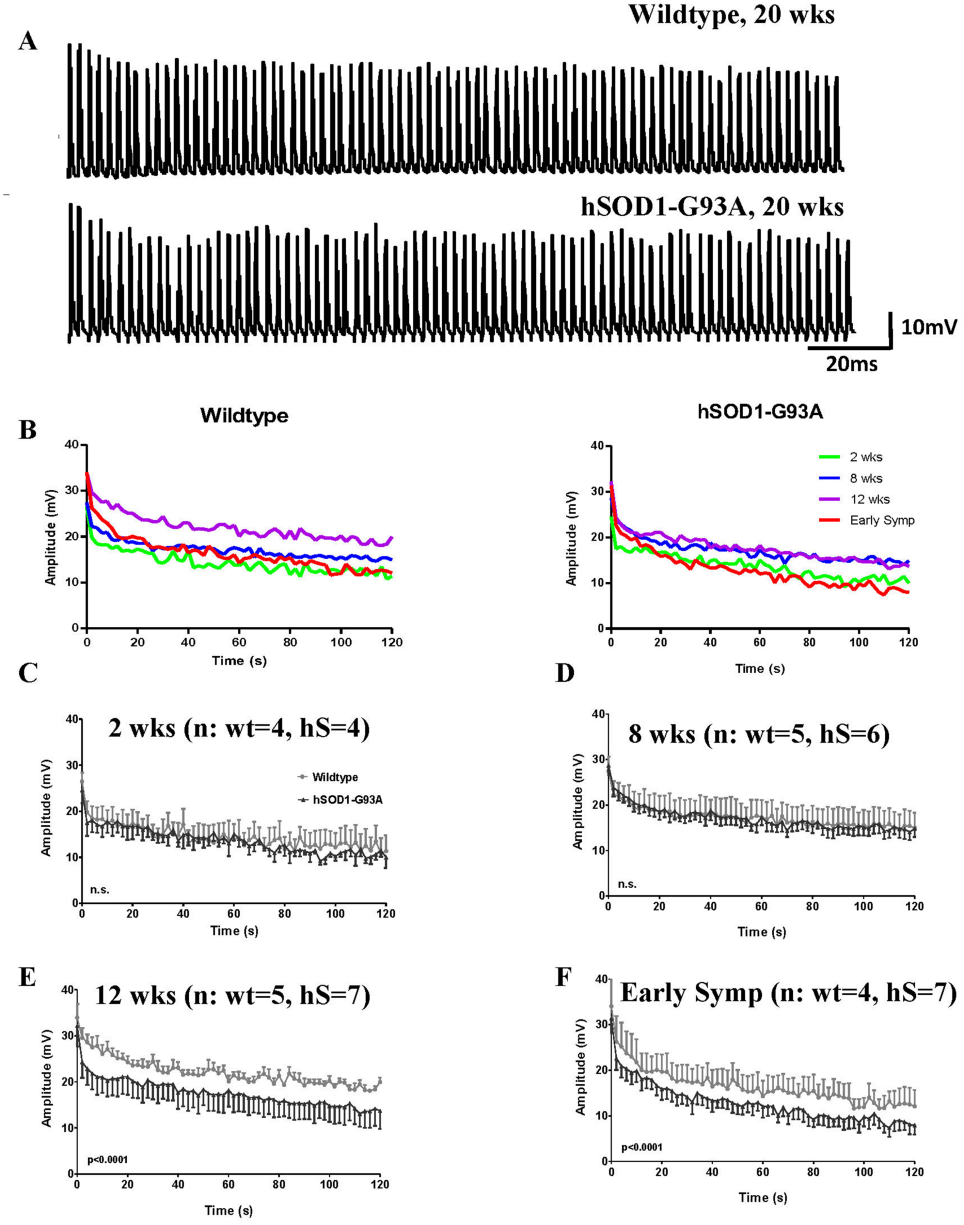
Maintaining release is impaired from 12 weeks at hSOD1-G93A mouse NMJs. **(A)** First 2 s of EPPs from a two-minute train from representative 20  weeks wildtype (top) and symptomatic hSOD1-G93A (bottom) mouse NMJs. EPP amplitudes decrease with number of pulses (‘rundown’), particularly during the first 10 EPPs of the train. **(B)** Mean EPP rundown comparison with age for wildtype (left) and early symptomatic hSOD1-G93A (right). At wildtype NMJs, initial EPP amplitude increases and ability to maintain it improves until 12 weeks, then rundown increases in the oldest animals. Patterns are similar in hSOD1-G93A mice, except EPP amplitude and ability to maintain it do not increase between 8 and 12 weeks. **(C–F)** Direct age-matched comparison (mean ± SEM), showing increased rundown in hSOD1-G93A from 12 weeks of age (both *p* < 0.0001, two-way ANOVA).

Typical responses to the first 2 s of a 20 Hz stimulation train are shown for wildtype and early symptomatic hSOD1-G93A NMJ in Figure 4. Both showed a progressive decrease in EPP amplitude during the train of pulses, called ‘rundown’ (Toonen et al., 2006) While this is normal, excessive rundown makes it more likely the EPP will fall below threshold for activating muscle fiber contraction.

The ability to maintain EPP amplitude (Figure 4A) was compared between wildtype and hSOD1-G93A mice to determine at what age any impairment emerged. In wildtype NMJs, as seen previously in the single stimulus responses, initial EPP amplitude increased up to 12 weeks then stabilized (Figures 4B–E). By 20 weeks (the oldest age), even in healthy wildtype mice, the ability to maintain EPP amplitude was reduced compared to younger mice. In hSOD1-G93A mice up to 8 weeks old, rundown was not significantly different to wildtype mouse NMJs (*p* > 0.05, Two-way ANOVA; Figures 4B–D). However, rundown in EPP amplitude became significantly greater at 12 weeks old (*p* < 0.0001, Two-way ANOVA; Figure 4E), 6 weeks before the overt motor symptoms appeared *in vivo*. The reduction in ability to maintain EPP amplitude then continues into the early symptomatic hSOD1-G93A stages (Figure 4F; *p* < 0.0001, Two-way ANOVA).

Thus, overall, NMJ deficits in the ability to maintain release (EPP amplitude rundown) in trains typical of *in vivo* activity appeared at 12 weeks, which is 6 weeks before obvious early motor symptoms.

### 3.3. Little gross morphological change, but significant postsynaptic AChR redistribution at diseased NMJs

The efficacy of neuromuscular transmission depends upon the strong structural correlation between the motor nerve terminal that releases the neurotransmitter and the postsynaptic ACh receptor (AChR) distribution with which the ligand interacts. Given these changes in presynaptic transmitter release, it was therefore important to determine if they were accompanied by changes in the AChR distribution postsynaptically. Thus, the AChR distribution was next examined in these same muscles.

Immediately after electrophysiological recording, TRITC-labeled α-bungarotoxin was added to the muscle superfusate to label the AChR distribution at the NMJ. In healthy muscles, this postsynaptic AChR distribution closely reflects that of the overlying presynaptic terminal, although this was not visualized in the current experiments. However, changes to the AChR distribution would still indicate an impact of disease (Schaefer et al., 2005).

The postsynaptic morphology was analyzed using the NMJ-morph algorithm (Jones et al., 2016), fractal analysis of NMJ perimeter and AChR label intensity distribution across the NMJ.

While it is known that NMJs become denervated during ALS, and axon retraction bulbs have been demonstrated as the endpoint of that process (Schaefer et al., 2005; Alhindi et al., 2022), the initial stages of this process have not been intimately studied. If the motor impairment is overt only when total denervation of a substantial number of NMJs has occurred, this would need a qualitatively different therapeutic intervention than if disease occurred with globally reduced function, but NMJs are largely structurally intact. We were examining how the earliest stages of disease manifest, so at least the great majority of the NMJs were innervated. I.e., it was not noticeably more difficult to find mEPPs in hSOD1-G93A muscles in the early symptomatic stages. Total and rapid denervation causing muscle fiber paralysis has little effect on AChR distribution, whereas ongoing muscle activity with retraction of individual nerve terminals boutons causes rapid AChR re-organization (Balice-Gordon and Lichtman, 1990; Balice-Gordon and Lichtman, 1993; Bishop et al., 2004; Tapia et al., 2012). Considering ALS as a ‘die-back’ disease (Dadon-Nachum et al., 2011), there are a number of possible ways, therefore, in which it might be proposed that AChR distributions can be morphologically disrupted as disease causes terminals to fail:

1. No disruption (terminal is abruptly removed without redistribution of the AChRs).
2. A global change in end-plate area or shape.
a. Endplates decrease in size (e.g., global nerve terminal retraction from the periphery toward the pre-terminal axon triggers removal of AChRs from peripheral non-innervated areas).
b. Endplates, at least initially, increase in size (e.g., compensatory nerve terminal growth, to increase synaptic area to offset terminal functional deficiency).
3. Local changes in end-plate shape and AChR distribution.
a. Fragmentation (small regions of AChRs are withdrawn, reflecting terminal bouton removal in a piece-meal fashion).
b. Local or global redistribution of AChRs across the whole endplate (AChRs occupy the same area but there is spatial redistribution).

These categories, of course, may each occur but at different times. However, it might be useful clinically to identify a particular characteristic as a biomarker of disease progression, perhaps in motor point biopsies. To try to understand which changes occurred and when, 10 postsynaptic morphological parameters were quantified from 4 weeks to early symptomatic ages: (see Methods for details). Many of these data were not normally distributed, so all were analyzed by non-parameteric statistical methods.

**Figure 5:**
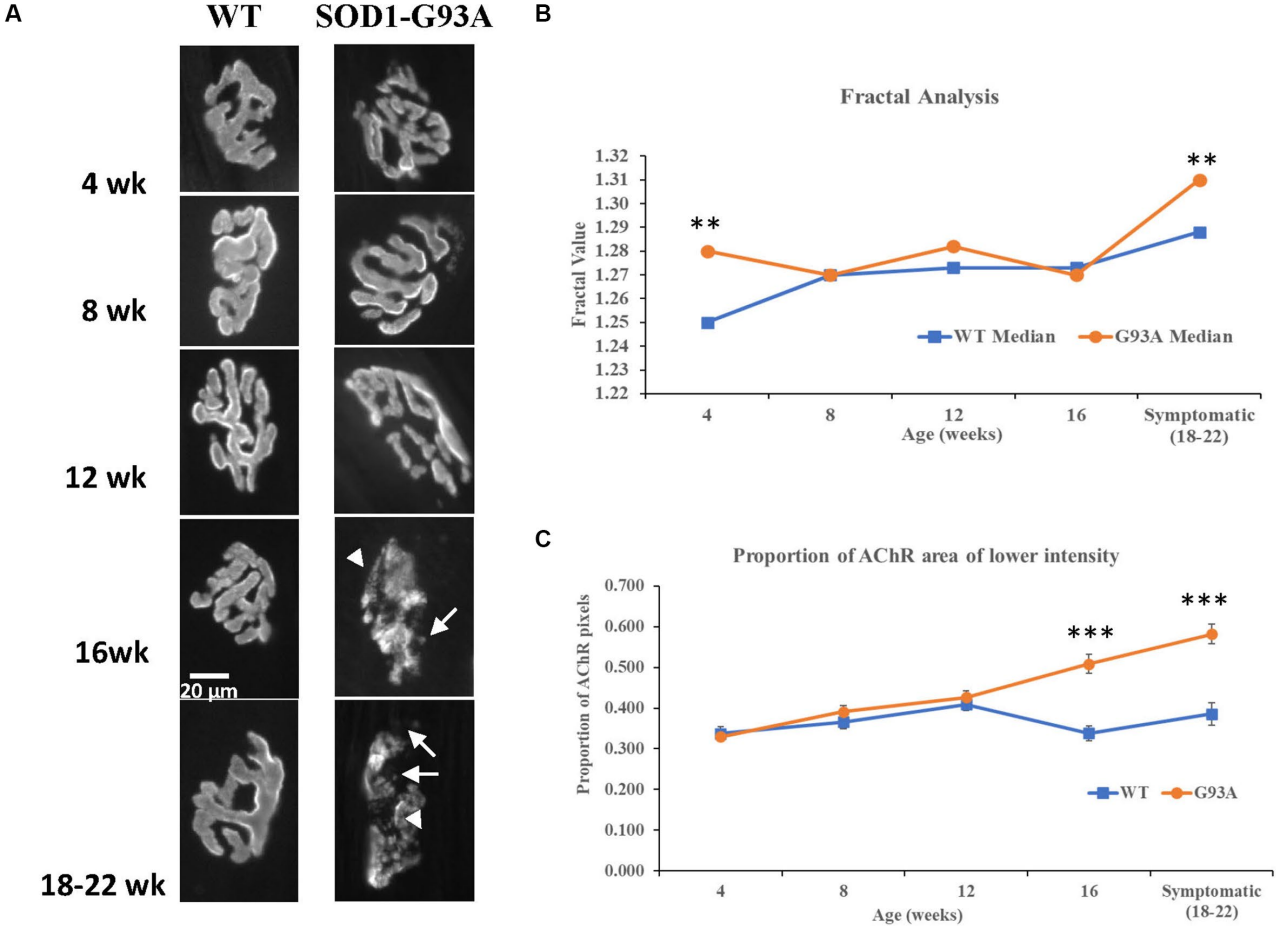
AChR perimeter becomes more complex, and AChR expression is disrupted with disease progression. **(A)** Representative AChR distributions at NMJs at each postnatal age. Healthy AChR distributions generally had relatively uniform bright ribbons in a typical complex ‘pretzel’ shape, that are known to well-represent the distribution of the overlying motor terminal. AChR ribbons had clear, crisp borders, within which AChR labelling was of relatively uniform intensity.Where detail is seen (e.g. WT at week 16, see also Figure 3B) the AChR distribution is a linear array, resembling finger prints and reflects the pattern of the postsynaptic membrane folds. At wildtype NMJs, this pattern is typically seen throughout the age range. In hSOD1-G93A mice, abnormalities appeared around 12 weeks and became increasingly common. By 16 weeks and early symptomatic stages (18-22 weeks), AChR distributions began to adopt a granular appearance (arrowheads), and crisp borders were typically lost. There was an increasing incidence of AChR spots beyond the borders, possibly reflecting new AChR expression under terminals sprouts, or remnants of extensive AChR removal (arrows). **(B)** Fractal analysis of the AChR perimeter showed a slowly increasing complexity with age at wild type NMJs. At hSOD1-G93A NMJs, the complexity was significantly higher at 4 weeks (**p = 0.008, MW), but stayed constant until 16 weeks, when it increased substantially, to become significantly greater than age-matched wild-type mice (**p = 0.009, MW). This suggests fragmentation of the NMJ perimeter with disease. **(C)** AChR expression levels were not different up to 12 weeks, with a very slowly increasing proportion of low AChR expression levels in both genotypes. However, while this situation stabilised at approximately 40% in wildtype mice from 12 weeks on, in hSOD1-G93A NMJs, the proportion of the NMJ with lower AChR expression increased progressively to 60% at the early symptomatic age (16 week and pre-symptomatic both ***p<0.001, Two-way ANOVA - Bonferroni post-test).

Images of typical AChR distributions at NMJs at each age are seen in Figure 5. The AChR distribution in the hSOD1-G93A NMJs appeared normal until 16 weeks. At 16 weeks and older, AChRs in some NMJs had a granular, rather than fingerprint, array with many small circular patches with lower or no AChRs. Interestingly, this coincided with the decrease in QC. However, when analyzed quantitatively, despite instances of significant differences in median values at individual age points (see Supplementary Data), none of the parameters displayed clear trends to be sustained or continually diverge from wildtype, or even show significant difference at early symptomatic ages (see Supplementary Data).

In general, parameters that increased with age were AChR area and perimeter, end-plate area, perimeter and diameter, plus area unoccupied by AChRs. Conversely, this produced reduced compactness and area per AChR cluster with age. The number of clusters and fragmentation was increased, only in the oldest age-group. However, as stated above, none of these showed sustained, statistically significant differences between genotypes, even at symptomatic stages. In contrast, the following parameters did show sustained and significant increases in variability from 12 weeks onwards: AChR area (*p* < 0.005 at 12, 16 and symptomatic; *F*-test) and number of AChR clusters (*p* < 0.21 at 12 weeks, *p* < 0.005 at 16 and symptomatic ages; *F*-test).

Thus, no individual parameter seemed a definitive biomarker of disease and/or its progression. We therefore examined further parameters which might fulfill such a role.

Fractal analysis of the AChR perimeter, might detect an increase in complexity if AChRs are remodeling. Conversely, the AChR area with lowered labeling intensity would indicate withdrawal of AChRs within a stable endplate perimeter. Fractal analysis of the end-plate perimeter (Figure 5B) revealed it generally increased with age in both genotypes. However, it had higher values in hSOD1-G93A mice only at the youngest (4 weeks, *p* < 0.01; MW, *n* = 42 and *m* = 4 wildtype vs. *n* = 79 and *m* = 9 hSOD1-G93A) and early-symptomatic ages (*p* < 0.01; MW, *n* = 67 and *m* = 12 wildtype vs. *n* = 42 and *m* = 4 hSOD1-G93A). Thus, hSOD1-G93A may reach mature AChR perimeter complexity early (higher fractal value at 4 weeks), then wild-type NMJs catch up, only for the fractal value to increase disproportionately at early symptomatic stages in hSOD1-G93A mice, when remodeling might be expected to increase.

The most robust indicator of NMJ structural disruption turned out to be the proportion of each NMJ with lower AChR labeling intensity. As shown in Figure 5C, from 16 weeks NMJs in hSOD1-G93A mice had larger areas with a significantly lower AChR intensity labeling, indicating reduced AChR expression (*p* < 0.0001, wildtype *n* = 30, hSOD1-G93A *n* = 45, Bonferroni post-test). This disruption increased with disease progression (*p* < 0.0001; 18–22 weeks, wildtype *n* = 29, hSOD1-G93A *n* = 51, Bonferroni post-test), whereas it plateaued in the wildtype NMJs from 12 weeks.

**Figure 6:**
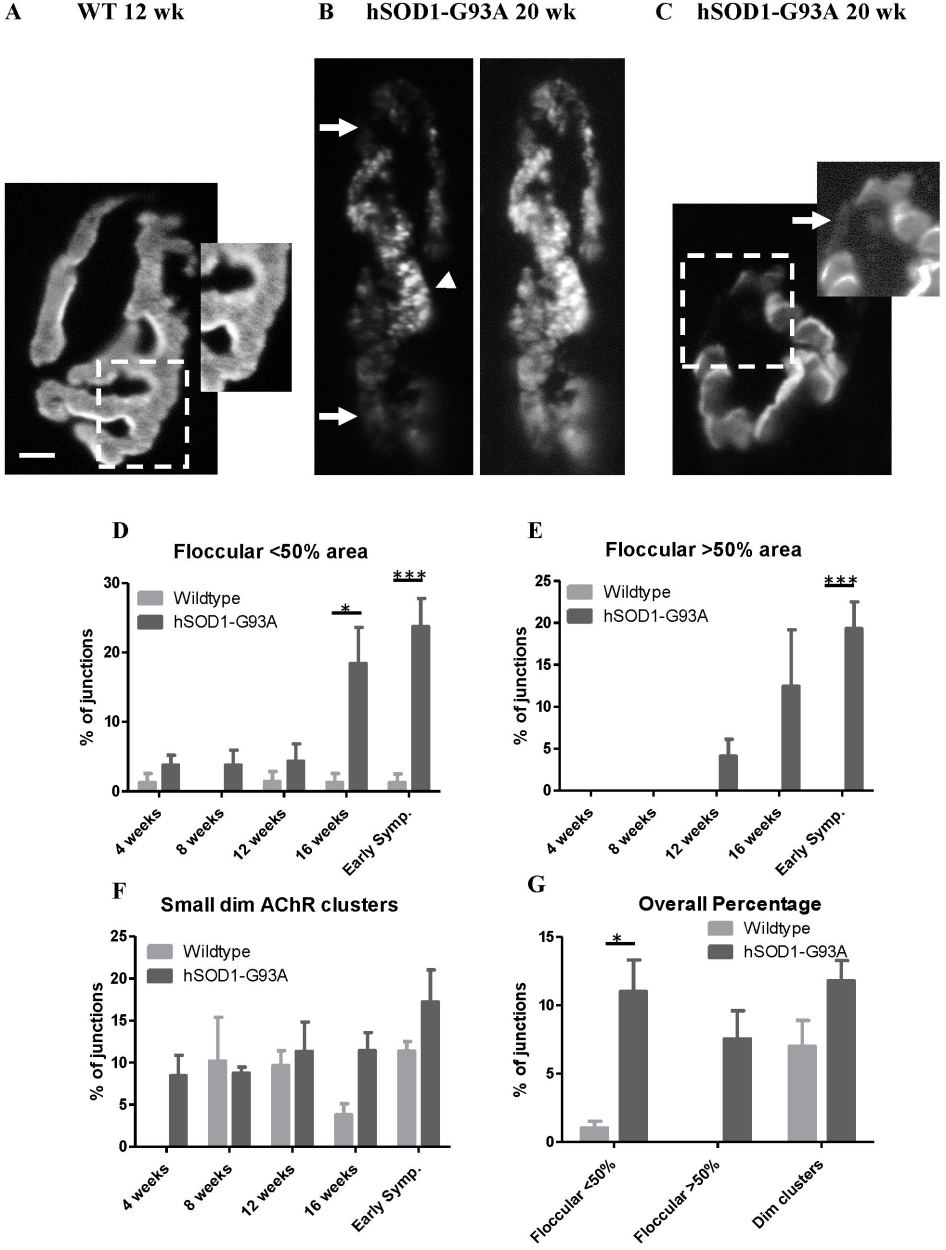
Disrupted AChR distribution with age at NMJs in hSOD1-G93A. **(A)** The labeling intensity of AChR labeling with TRITC-BgTx at this 12 weeks. wild type NMJ is typically very uniform, with a crisp border. The inset of a magnified and enhanced region shows the typical finger-print distribution of labeling at the crests of the postsynaptic folds. **(B)** One of the more disrupted NMJs from a symptomatic (20 weeks) hSOD1-G93A. A floccular/granular AChR label distribution (arrowhead) has replaced the normal finger-print pattern, and much of the NMJ is dimmer than normal (arrows), shown more clearly in the enhanced image (right) of the same NMJ. **(C)** A less affected NMJ from another early symptomatic hSOD1-G93A mouse, with only a small region of disrupted very dim AChRs (boxed area), indicating early remodeling. Enhancing the image (right inset) shows the small, dimly labeled AChR clusters (arrow) either being inserted or removed. Scale bar = 10 μm (main images) or 2 μm (inset in **A**). **(D)** In wild-type NMJs, the incidence of floccular, rather than fingerprint-like, distribution in small areas is low and consistent across all ages. In hSOD1-G93A, the incidence tended to be higher overall (*p* < 0.001, two-way ANOVA). The incidence in these mice increased markedly at 16 weeks (*p < 0.05, Bonferroni post-test), and further into early symptomatic stages (***p < 0.001, Bonferroni post-test). **(E)** No NMJs in wildtype muscles had a floccular AChR distribution extending over >50% of the area, unlike hSOD1-G93A mice overall (*p* < 0.01, two-way ANOVA). In the latter, they were first seen at 12 weeks after which the incidence progressively increased, reaching over 20% at the age first motor symptoms appeared (***p < 0.001, Bonferroni post-test). **(F)** Approximately 10% of NMJs displayed small, dim clusters of AChRs across all ages and genotypes, although they appear earlier in hSOD1-G93A mice. **(G)** Summary of incidence of NMJs with each of these parameters across the age range.

Overall, this analysis indicated there was no large-scale disease-driven shrinkage or growth in the footprint of the NMJ. Rather, there was a re-distribution of AChRs within the original footprint. We therefore then undertook a more detailed examination of AChR patterns within the NMJ. In wildtype NMJs, where detail of AChR distributions were seen, this was normally in ‘fingerprint-like’ linear arrays, reflecting concentrations at the crests of postjunctional folds (Figure 6A; Flucher and Daniels, 1989). In NMJs where image resolution was insufficient, this AChR distribution looked essentially uniform intensity within the ribbons, with a crisp border to each ribbon (Figure 5). In hSOD1-G93A, from 12 weeks onwards, this appearance was gradually replaced by a more obviously granular distribution and loss of crisp borders (Figure 6B). Increasingly, some NMJs also developed small islands of AChRs, usually much dimmer than the main AChR ribbons, and lying beyond the normally smooth NMJ perimeter (Figures 6B,C).

These two parameters (floccular AChR distribution and small dim extrajunctional clusters) were quantified in 20 NMJs from each muscle of 2–5 mice at each age (see Methods).

Small floccular AChR regions were seen at an essentially constant rate of <5% in wildtype NMJs across all ages (Figure 6D). There was no trend for this incidence to increase with age in healthy mice. In hSOD1-G93A mice, up to 12 weeks, the incidence was initially similar. However, at 16 weeks there was a marked increase in the incidence of floccular AChR regions, and it progressed to cover increasingly larger areas of the NMJ in symptomatic animals. NMJs with >50% of the area of granular AChR distribution (Figure 6E) were only seen in hSOD1-G93A. Such grossly affected NMJs first appeared at 12 weeks. This corresponded with the first detection of impaired ability to sustain release in repetitive trains, and significantly shorter EPP rise times. By the time of the appearance of motor symptoms, the incidence of AChR disruption had risen to a mean of >20% of NMJs examined. There was, however, no systematic significant difference in the incidence of small, dim extrajunctional clusters between wild-type and hSOD1-G93A overall (Figures 6F,G).

## 4. Discussion

This study aimed to understand the physiological and anatomical condition at NMJs when the first, mild motor impairments become detectable in ALS. We used hSOD1-G93A mice, the most widely used model of ALS. By understanding this, we aimed to gain an insight into the level of disruption likely to be present when ALS patients first present to the clinician. In motor terms, for these mice, the proxy here was the appearance of tremor in the hindlimbs when the hind quarters were elevated clear of the gound by the tail. By charting NMJ morphology and neurotransmission throughout postnatal development, this study also aimed to identify how many identified disruptions were present before *in vivo* motor symptoms emerge, and their extent. Characterizing this could give invaluable insights into how long the underlying processes have been developing and, thereby, indicate what any treatment might need to overcome even at these earliest clinical stages. Finally, correlation of electrophysiology and morphology in the same muscle preparation aimed to indicate the likely contribution, and nature, of structural disruption to the physiological impairment. Overall, therefore, we aimed to understand the processes that might need to be corrected within NMJs that are still functional, although impaired, to offset symptoms. Therefore, this study aimed to give a clear timeline of functional and structural changes, their relative extent and contributions as the context within which therapeutic intervention would occur.

The hSOD1-G93A mice in our colony had a delayed onset of the condition (18–22 weeks, or 126–154 days) relative to original strain (Broadhead et al., 2022). Exactly why is unclear, but presumably this results from their re-derivation onto another genetic background (Pfohl et al., 2015). Diseased mice are heterozygote, as homozygotes either do not survive to breed or rarely produce viable pups. Thus, heterozygotic founder males were crossed with females of our in-house C57/Bl6 colony. It is also possible there was a reduction in copy number of the hSOD1-G93A during this process. Once established, however, the timing of developing this phenotype was stable. There was no further change in time to progress to point 3 on the Weydt scale (Weydt et al., 2003). Regardless, all genotyped hSOD1-G93A positive mice kept long enough went on to develop hindlimb tremor. Therefore, the progression seems likely to reflect the same disease process but over a more prolonged time course.

It is also possible that the functional and morphological deficits seen reflect an intrinsic difference in vulnerability to axotomy in the acute experiments used to make the observations, since a number of studies have examined, for example, Wallerian degeneration under similar conditions (Di Stefano et al., 2015; Kline et al., 2019; Mole et al., 2020). However, such studies were performed at higher temperatures (28–37°C), while control experiments at lower temperatures (25°C) reduced any effects such that they were undetectable on the timescale of the current experiments – at least for healthy axons. Thus, the possibility exists defects in the hSOD1-G93A reflect an enhanced sensitivity to axotomy in the ALS mice. Regardless, there are clear functional deficits that appear at NMJs in these mutant mice.

Overall, NMJ functional perturbations without repetitive stimulation were present from at least 8 weeks but were minor, even when significant, even in symptomatic animals. Specifically, spontaneous potentials (mEPPs) tended to be larger until 8 weeks, in agreement with a previous electrophysiological study of diaphragm NMJs from presymptomatic mice (Rocha et al., 2013). The amplitude then declined to ~79% of wildtype at early symptomatic ages. Impairments in single evoked responses (EPPs, QC) were only detected either just as motor symptoms emerged (EPPs are smaller at 18–22 weeks) or just before (smaller QC at 16 weeks, but transiently). Interestingly, we did not find the increased QC in hSOD1-G93A previously reported (Rocha et al., 2013). Although why is not clear, and both might be due to a number of mechanisms, it could be speculated that it reflects the loss of vulnerable motor neurones, with the more robust surviving at later dates, seen as an increase of QC. Alternatively, it may be that muscle fibers undergoing denervation initiate some compensatory feedback mechanism. Further studies would be needed to determine the precise mechanism. However, it was during the *in vivo*-like activity of maintained trains of stimulation that much more substantial, and progressive, deficits became apparent. Thus, EPP amplitude during 20 Hz trains of stimuli fell to ~50% of wildtype at the end of 2 min trains from 12 weeks of age, well before the emergence of overt motor symptoms. While we did not seek to determine the cause of this, two pathologies are known to be present that could be responsible. First, a shortage of vesicles available for release, due to a reduced vesicle density (Cappello and Francolini, 2017) and, second, shortage of ATP, since mitochondria show increased pathology at all stages in disease in hSOD1-G93A mice (Rogers et al., 2017). Given the sudden onset of this deficit at 12 weeks, it seems likely to be a reduced vesicle density, rather than a chronic impairment of mitochondrial function.

Similarly, there were no gross changes in morphological parameters at NMJs as determined by NMJ-morph, which aims to provide a robust analysis of the principal components of NMJ morphology. In essence, this analysis shows that the footprint of the AChR distribution changed little even by symptomatic stages, although it must be remembered that this was the earliest symptomatic stage, and there was a significant increase in variability of some parameters. The lack of change in detailed footprint parameters reported in the present study is in agreement with previous less-detailed studies (Schaefer et al., 2005; Mejia Maza et al., 2021), which found this to be true throughout the disease. Thus, for at least the endplate (postsynaptic) aspect of the NMJ, there is no robust evidence of shrinkage at this early stage. Rather, the most substantial and robust disruption was in AChR redistribution within the original NMJ footprint. Again, this accords with previous studies (Schaefer et al., 2005), where simultaneous presynaptic terminal imaging showed the granular areas are associated with local denervation. The first appearance of this disruption was as early as 16 weeks, which is 2 weeks before overt motor symptoms, but actually 4 weeks after the impaired ability to maintain release (seen at 12 weeks). Interestingly, it coincided with the transiently diminished QC, suggesting the reduction in vesicles per EPP might be correlated with, even causative in, inducing the rearrangement. There were two aspects of AChR redistribution identified: diminished expression (lower intensity) and disrupted pattern (floccular appearance) of AChRs. These were both much more robust structural markers of disease than changes in overall NMJ shape. This again accords with the denervated/faint AChR NMJs reported by (Schaefer et al., 2005) in this muscle. It seems likely the changes in AChR distribution reflect some combination of withdrawal of pre-existing AChR clusters, and the disruption of postsynaptic folds reported by Cappello et al. (2012) and Vinsant et al. (2013). It may also reflect expression of embryonic AChRs, which tend not to label so well with α-bungarotoxin (personal observations). Again, this may reflect the altered expression of agrin and LRP4 at NMJs in hSOD1-G93A (Clark et al., 2016). The separated small, dim areas likely reflect insertion of new AChR clusters under sprouting presynaptic motor neuronal terminal, while those close to the AChR ribbons may rather be vestiges left behind as the surrounding AChRs are in the process of being withdrawn. Schaefer et al. (2005) found very little evidence of terminal sprouting when they examined terminal and AChR distribution at the same NMJs, finding where present, that most sprouts originated from the axon. They also found terminals underwent fragmentation when degenerating, rather than retraction. Again, this is consistent with the lack of change in shape and size of the NMJs.

Finally, the percentage of NMJs showing impaired ability to maintain release during trains at early symptomatic stages was very widespread. Although not examined systematically, it was certainly the great majority impaired in this way. That is, most nerve terminals, perhaps all, had functional impairment at early symptomatic stages. In contrast, only ~20% of NMJs had >50% of their area appearing floccular (= denervation-like) appearance, with another ~20% at <50%. If the latter are only partially denervated, this implied that as many as 80% of NMJs may retain some structurally intact innervation and function at this stage.

Thus, overall, substantial functional disruption, i.e., an inability to maintain release, occurred 6 weeks before the earliest motor symptoms, followed 4 weeks later by floccular redistribution and reduced expression of AChRs within the NMJs, which likely reflects the onset of denervation. This occurs 2 weeks before overt motor symptoms appeared. There was never a substantial NMJ shape change. Rather, >20% of NMJs developed floccular or granular AChR distributions, and > 50% had developed significant areas of the NMJ with reduced AChR labeling density. These observations indicate, therefore, that the functional changes precede the AChR redistribution and, therefore, the motor terminals are impaired for a significant period before AChR redistribution. It seems likely that at least 80%, and possibly as many as 90% (Schaefer et al., 2005) of NMJs have substantive innervation but impaired function at the onset of overt symptoms. This indicates the major deficit at the stage of clinical presentation in patients is likely to be reduced functionality rather than widespread denervation. Thus, therapies targeted at supporting transmission and reversing impairment in motor neurone health seem most appropriate to most significantly impact on relieving symptoms of ALS at these early stages. For example, it might be worth investigating the use of anticholinesterases, as is common practice for myasthenia gravis, such as pyridostigmine or neostigmine, perhaps with muscarinic side-effects being antagonized by atropine or propantheline.^2^ Alternatives might be repurposing drugs used for Alzheimer’s disease, such as donepezil, rivastigmine or galantamine (Birks, 2006). Another option might be targeting endogenous modulatory systems, such as TGF-β2 (Fong et al., 2010), neurotrophins (Harandi et al., 2016) or peripherally restricted cannabinoids (Newman et al., 2007). As well as directly supporting neurotransmission, such approaches that improve muscle activation could enhance the activity-dependent release from active muscles of a cocktail of other factors supportive of motor neurone survival (Saini et al., 2021).

## Data availability statement

The original contributions presented in the study are included in the article/Supplementary material, further inquiries can be directed to the corresponding author.

## Ethics statement

The animal study was reviewed and approved by University of Aberdeen, Animal Welfare and Ethical Review Board.

## Author contributions

JM, GB, and GM contributed to conception and design of the study. JM, IM, MD, CG, and RB performed the data analysis and statistical analysis. JM wrote the first draft of the manuscript. GB, GM, IM, MD, CG, and RB wrote sections of the manuscript. All authors contributed to manuscript revision, read, and approved the submitted version.

## Funding

This project was funded by a PhD studentship to JM from Motor Neurone Disease Scotland, on grant number RGB3753 to GB and GM.

## Conflict of interest

The authors declare that the research was conducted in the absence of any commercial or financial relationships that could be construed as a potential conflict of interest.

## Publisher’s note

All claims expressed in this article are solely those of the authors and do not necessarily represent those of their affiliated organizations, or those of the publisher, the editors and the reviewers. Any product that may be evaluated in this article, or claim that may be made by its manufacturer, is not guaranteed or endorsed by the publisher.

## Supplementary material

The Supplementary material for this article can be found online at: https://www.frontiersin.org/articles/10.3389/fnmol.2023.1169075/full#supplementary-material

## Footnotes

^1^ https://imagej.nih.gov/ij/

^2^ https://bnf.nice.org.uk/treatment-summaries/neuromuscular-disorders/#drugs-that-enhance-neuromuscular-transmission
