# Supplementary figures and images for: Development of abnormalities at the neuromuscular junction in the SOD1-G93A mouse model of ALS: dysfunction then disruption of postsynaptic structure precede overt motor symptoms
Source: Front Mol Neurosci. 2023 May 19;16:1169075. doi: 10.3389/fnmol.2023.1169075 (PMC10237339; doi:10.3389/fnmol.2023.1169075)

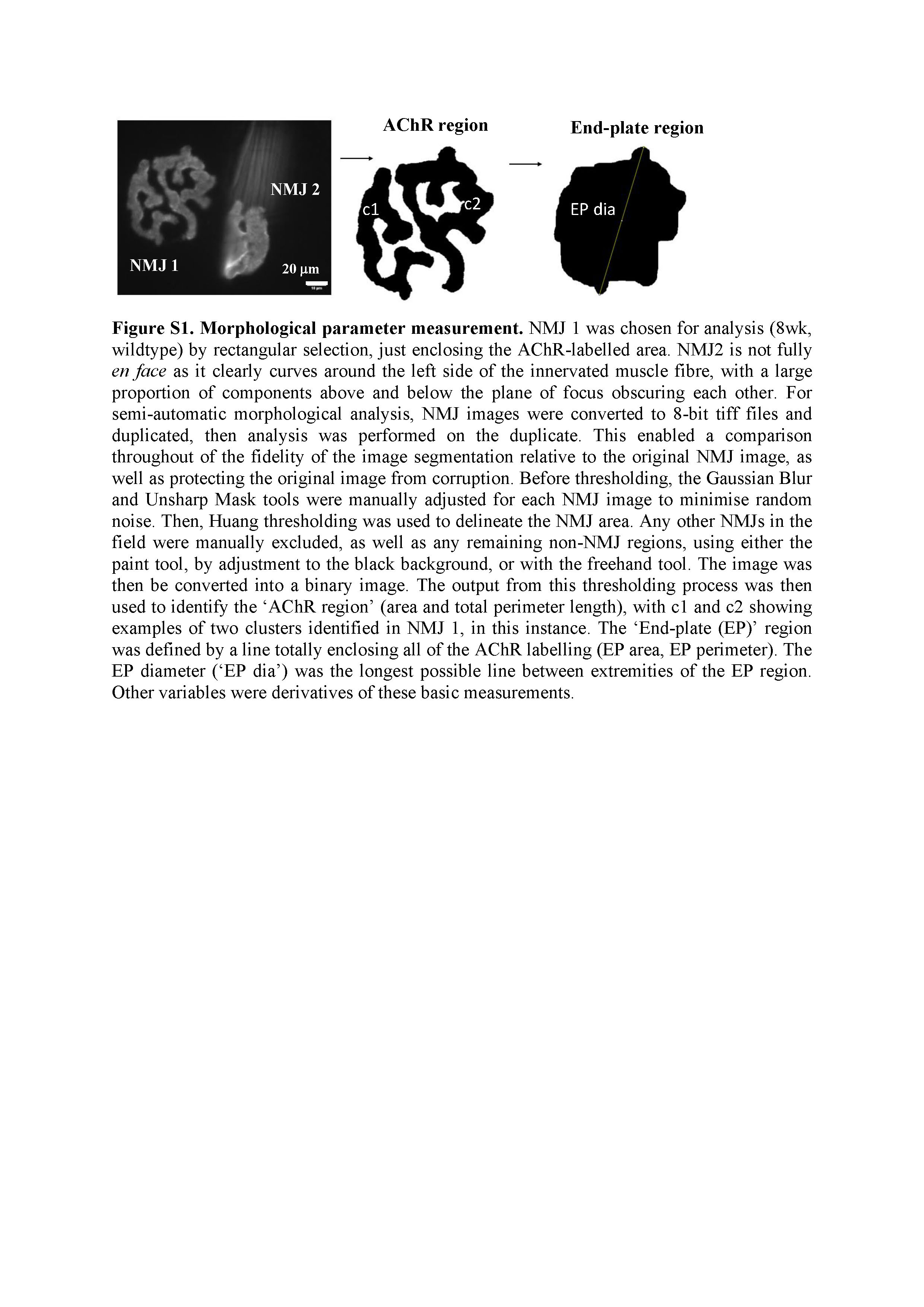

Supplement: Supplementary file 2 [file Image_1.JPEG]

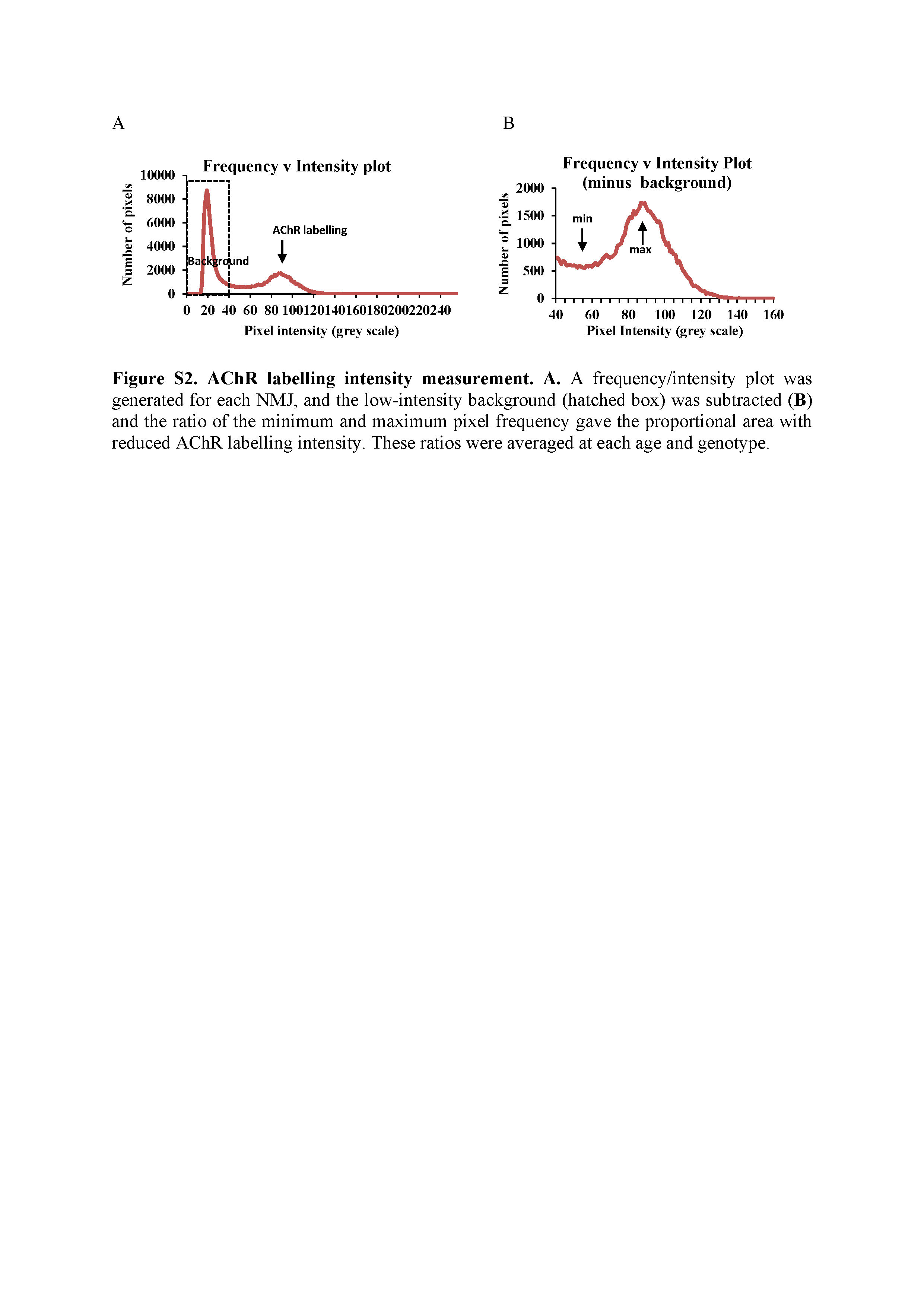

Supplement: Supplementary file 3 [file Image_2.JPEG]

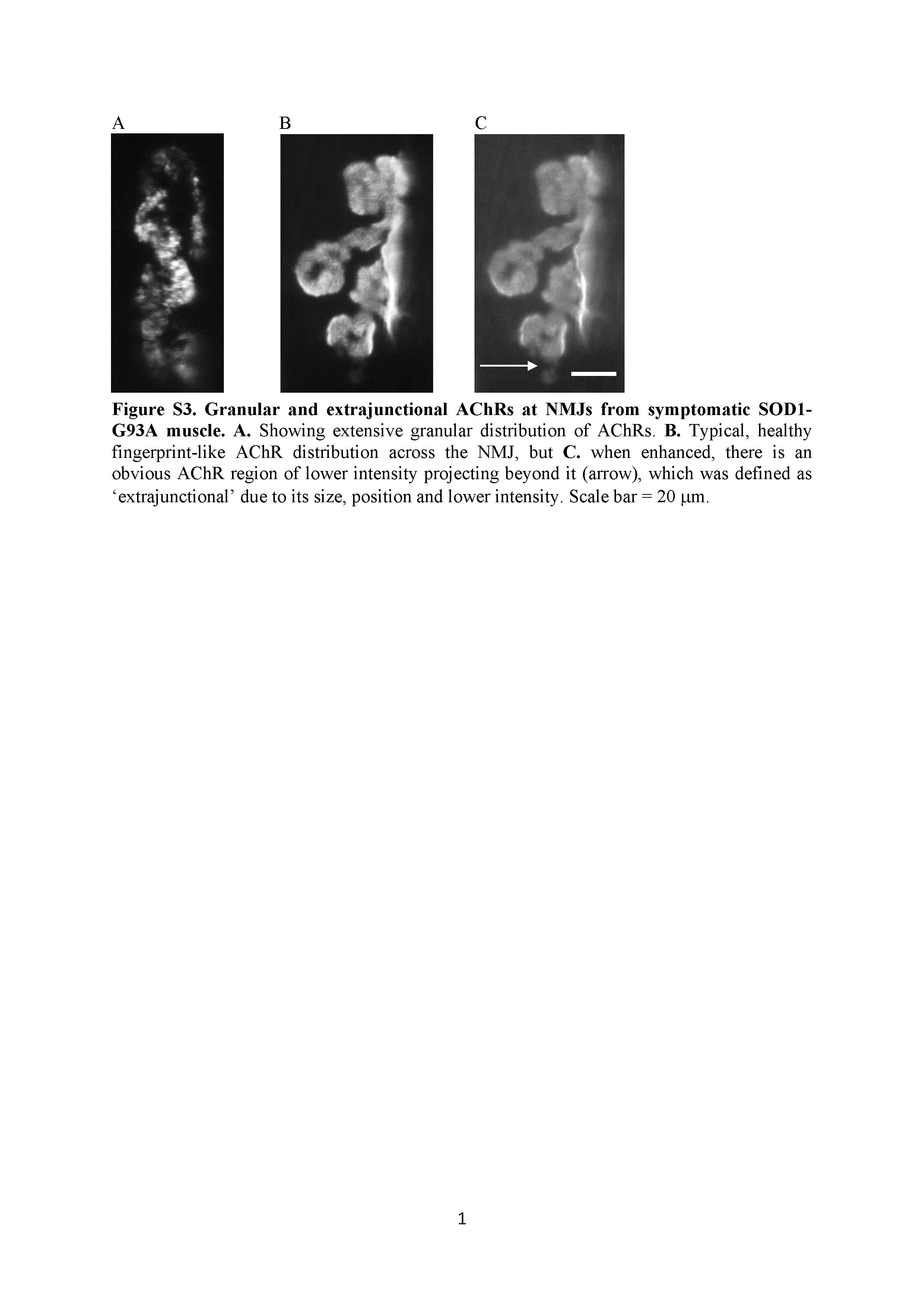

Supplement: Supplementary file 4 [file Image_3.JPEG]
